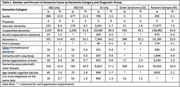# Examining Dementia Subtypes in a National Sample of Autistic Medicaid and Medicare Enrollees

**DOI:** 10.1002/alz70860_100521

**Published:** 2025-12-23

**Authors:** Lindsay Shea, Giacomo L Vivanti, Wei‐Lin L Lee, Jonas L Ventimiglia, Teal Benevides, Laura N. Gitlin, Laura Klinger, Kristen L Lyall, Brian L Lee, Nancy R Lee, Jessica L Rast, Diana L Schendel, Gregory L Wallace

**Affiliations:** ^1^ Drexel University, Philadelphia, PA, USA; ^2^ Augusta University, Augusta, GA, USA; ^3^ University of North Carolina, Chapel Hill, NC, USA; ^4^ George Washington University, Washington D.C., DC, USA

## Abstract

**Background:**

Small sample sizes have constrained the examination of dementia subtypes in Autism Spectrum Disorder (ASD) compared to other groups. Data from Medicaid and Medicare, which enroll tens of millions of Americans, can address this limitation and support identification of system‐level improvements to address needs of those with ASD and co‐occurring dementia. This study sought to examine dementia subtypes among autistic individuals compared to other groups.

**Method:**

Individually‐linked national Medicare and Medicaid data from 2014–2016 included individuals age 30 and older with dementia from mutually exclusive groups with ASD (2,229 with no intellectual disability (ID), 4,263 with ID), 52,198 with ID, 1,199 with Down Syndrome (DS), and a random sample of 237,009 individuals without these diagnoses (RS). The validated Bynum algorithm was organized into the subtypes shown in Table 1. The last observed dementia diagnosis was used for dementia subtype. Following preliminary analyses showing similar data for those with ASD with and without ID, a single ASD category (ASD only and ASD+ID) was used.

**Result:**

Unspecified dementia represented the largest proportion of cases for all groups (50.0% for ASD, 56.3% for ID, 42.1% for DS, and 54.3% for RS). The ASD group had the lowest proportion of Alzheimer's disease cases (11.7%), compared to ID (14.2%), DS (32.2%), and RS (22%). Senile dementia represented a larger proportion of ASD dementia cases (11.5%) than the other categories combined (ID=1.5%, DS=0%, RS=0.1%). The relative proportion of other dementia subtypes did not differ substantially between ASD versus ID.

The proportion of all dementia subtypes among autistic females was an average of 25% lower than females in RS. A lower proportion of all dementia cases in non‐white adults was observed for ASD compared to RS.

**Conclusion:**

Examination of dementia subtypes suggests a lower proportion of Alzheimer's disease and a higher proportion of Senile dementia in ASD compared to other populations under examination, whereas the proportion of other dementia subtypes was generally aligned with that of participants with ID. Non‐white adults might be misdiagnosed or underdiagnosed in the ASD population, pointing to the possibility of ASD‐specific service implications amid the backdrop of rising dementia cases nationally.